# Baccalaureate nursing students’ experiences of how the COVID-19 pandemic influenced learning – a mixed method study

**DOI:** 10.1186/s12912-022-00955-6

**Published:** 2022-07-01

**Authors:** Gudrun Rohde, Berit Johannessen, Markus Maaseide, Sylvi Flateland, Anne V. Skisland, Ellen B. Moi, Kristin Haraldstad

**Affiliations:** 1 Department of Health and Nursing, Faculty of Health and Sport Sciences, University of Agder, Postbox 422, 4604 Kristiansand, Norway; 2 Department of Clinical Research, Sorlandet Hospital, Kristiansand, Norway

**Keywords:** Baccalaureate nursing students, COVID-19 pandemic, Learning, Mixed method

## Abstract

**Background:**

The COVID-19 pandemic mandated the reconstruction of educational programs globally. For nursing students in need of practical learning and training as a part of their curriculum, the pandemic also caused restrictions and alterations in practical placements and limited access to simulation training at campuses. The aim of this study is therefore, to describe and explore how the COVID- 19 pandemic influenced baccalaureate nursing students’ experiences of learning and their social life as a student.

**Method:**

We used a mixed method study design including quantitative data from one of the universities (University of Agder) from a national Norwegian survey of baccalaureate nursing students and qualitative data from focus-group interviews from the same university. The quantitative survey included 396 baccalaureate nursing students (response rate, 46%). We arranged five focus groups with a total of 23 students (15 females and 8 males). The students took part in one of the focus-group interviews. The quantitative data were analyzed using chi-square test for categorical data and ANOVA tests for continuous data, while the qualitative data were analyzed using Systematic Text Condensation and an editing analysis style.

**Results:**

Most students (70%) were concerned about the quality of the education program. In clinical placement, 83% of students had cared for patients with confirmed COVID-19 or unclear COVID-19 status. The qualitative data revealed three main themes: missing the social dimension of learning; worries and challenges in clinical placement; and experiencing normal instructive days in clinical placement. The general picture emerging from our analysis is the importance of social interactions for learning.

**Conclusion:**

As the COVID-19 pandemic continues, our findings of students’ worries about the quality of the education program and not reaching learning outcomes must be taken into consideration. Social interaction is considered crucial and fundamental for student learning. A follow-up study following baccalaureate completion is necessary to explore the consequences over time of disrupted education.

**Supplementary Information:**

The online version contains supplementary material available at 10.1186/s12912-022-00955-6.

## Introduction

In March 2020, the impact of the COVID-19 pandemic enforced the reconfiguration of educational programs globally. Online distance learning, digital seminars, and supervision became the new normal [1–4]. For students in need of practical learning and training as a part of their curriculum, restrictions and alterations in practical placements and limited access to simulation training at campuses became the new normal and threatened the viability of students’ education [2, 5–7]. Both students and professors had to adapt to the new situation [8].

Nursing students may be particularly vulnerable during a pandemic, since practical and clinical training is an important part of their study where they might meet patients with COVID-19 or unclear infection status. In clinical placements, they become part of the health care system and are exposed to stressful factors, such as fear of being infected and spreading the virus to patients [9–12]. Caring for critically ill COVID patients may be challenging and may cause anxiety and stress among nursing students [13–15]. Recent studies have shown that the COVID-19 pandemic has caused a variety of challenges to nursing students globally such as uncertainty, stress, problems with concentration and learning efficiency, and fear of infection. Students were also concerned about their grades, passing their exams, the final graduation [2, 11, 13, 14, 16–18], and their career ahead [11]. In addition, previous studies have identified loneliness, mental health problems, and sleeplessness among nursing students [11, 13, 15]. However, Swift et al. [17] also found that nursing students in the UK expressed a sense of being a part of history, learning new things, and stepping up to a challenge that will be personally fulfilling as well as professionally worthwhile.

Most nursing programs are traditionally conducted in a face-to-face learning environment and emphasize the importance of a sociocultural perspective on learning [19]. Students are regarded as active participants and co-constructors of learning, where learning is essentially a social term rather than individual in nature and where interaction constitutes the learning process [20]. A reduced or even absent possibility for interaction due to pandemic restrictions, and use of alternative platforms such as learning management systems and web conferencing, has changed the nursing students’ learning situation [1, 2].

At the current stage of the COVID-19 pandemic, most Norwegian nursing students as well as the general population are vaccinated. While statistics indicate that Norwegian society has handled the pandemic rather well, a new wave of coronavirus has arrived, and nurses and other health care providers have experienced a work overload and are exhausted [21, 22]. Most inhabitants trust the governmental rules and recommendations, the transmission rate has been relatively low, there have been few hospital admissions and few deaths, and the rate of vaccinated people is high compared with other countries [21]. Despite this, Norwegian baccalaureate nursing students have been affected by restrictions depending on local and temporal variations in the transmission rate. Practical training, which is normally 50% of the three-year baccalaureate nursing program (a total of 180 European Credit Transfer System points), has proceeded with modifications [23]. During the second wave of the pandemic (January–February 2021), a national survey including five Norwegian universities showed that nursing students’ academic concerns and satisfaction with the educational curriculum were significantly influenced by the pandemic, with the level of satisfaction being lower than pre-pandemic values [23]. The national survey data for baccalaureate nursing students’ experiences of the learning situation exist for the five Norwegian universities, and revealed some differences between the universities, most likely due to variation in the number of COVID-19 cases and restrictions in the regions [23]. However, in-depth knowledge alongside survey data seems to be lacking. The aim of this study therefore is; to describe and explore how the COVID-19 pandemic influenced baccalaureate nursing students’ experiences of learning and their social life as a student.

## Methods

### Design and sample

We used a mixed method study design including quantitative data from one of the five universities (University of Agder) from the national survey [23], combined with qualitative data from the same university. In the Southern part of Norway where University of Agder is situated the transmission rate was rather low, and the university was able to offer the student clinical placement nearly as normal. This was not the case for several the other universities. The quantitative and qualitative data were collected sequentially [24]. Inferences of each strand were integrated into meta-inferences at the end of the study and are presented in the Discussion section.

### Quantitative data

Sample: Between 27th January and 28th February 2021, full- and part-time baccalaureate nursing students > 18 years of age from the two campuses at University of Agder were invited to take part in a web-based cross-sectional survey together with those from four other universities in Norway.

The survey for the quantitative data included questions related to students’ demographics, personal health, and study situation during the pandemic, and was specifically developed for the present research by an expert group consisting of clinicians, nursing students, university staff, and researchers. By the time of the survey, second-year and third-year students had attended 1–3 periods of clinical practice during the COVID-19 pandemic, whereas first-year students had not.

**Characteristics of the respondents** included age (< 25, 25–29, ≥ 30 years), household status, study site, and year of study.

**The Fear of COVID-19 Scale (FCV-19S**) [25], which had been adapted and assessed for use with Norwegian samples, was used [26]. Seven items (e.g., “I am most afraid of the coronavirus”) were rated on a 5-point scale from 1 (strongly disagree) to 5 (strongly agree), with a total score ranging from 7 to 35. Higher scores represent a greater fear of COVID-19. In the present study, the average item score was used. It was calculated by dividing the total score by the number of items.

**COVID-19-specific questions related to personal health** were developed for the national study and included quarantine history (never, previous, present); feelings of loneliness due to COVID-19 (rated from 1 [strongly disagree] to 5 [strongly agree]); perceived risk for complications of COVID-19 (no, uncertain, yes); history of suspected, possible, or confirmed COVID-19 infection; and trust in authorities’ and universities’ handling of the pandemic (rated from 1 [strongly disagree] to 5 [strongly agree]). In the comparative analysis, agree and strongly agree were pooled as agreed.

**COVID-19-specific questions related to education** addressed students’ perceived impact on the different aspects of their education, especially the impacts on clinical practice and placements and the students’ concern about the quality of education.

The COVID-19-spesific questions related to the personal health and education were piloted with 9 nursing students (including MM, one of the authors of this study), and after minor adjustments, a digital pilot study was conducted with 90 physiotherapy students [27]. No adjustments were made after the digital pilot.

Statistical analysis: Descriptive statistics are presented for all students and also for the students within the different years of education specifically. Categorical variables are described as counts and percentages and continuous variables as means (standard deviation [SD]), Associations between variables were assessed using the chi-square test for categorical data and the ANOVA tests for continuous data [28]. All tests were two-sided. *P*-values < 0.05 were considered statistically significant. Furthermore, the analyses were considered exploratory, so no correction for multiple testing was performed. All analyses were performed using IBM SPSS Statistics (version 26) [28].

### Qualitative data

All three-year baccalaureate nursing students from both campuses of University of Agder were invited by the student representatives and the students learning platforms to participate in focus-group interviews in person, on campus. The focus-group interviews took place between 26th April and 6th May 2021.

We arranged five focus groups with a total of 23 students (15 females and 8 males), each comprising two to seven students. The students took part in one of the focus-group interviews arranged in the afternoons, lasting for 50–90 minutes. The interviews were moderated by the first author GR (four groups) or the last author KH (one group), both nurses and professors in health sciences. At the time of the focus-group interviews, all students had attended 1–3 periods in clinical placement during the pandemic.

In focus groups, people with similar experiences gather together to discuss a given topic, and conversation between the participants is central [29]. The dynamics between the participants allow for insights of a different nature from those obtained through individual interviews. We used a semi-structured interview guide to ensure the inclusion of the issues in focus. The students were asked questions such as: “How much do you think that the COVID-19 pandemic has influenced your learning and learning outcomes?,” “How did you experience that your placement was prepared for the COVID-19 pandemic,” and “How did this influence your learning?”

Qualitative analyses*:* We audiotaped the interviews and transcribed them verbatim. The interviews were transferred to NVivo V.12 software, to organize the transcripts into codes and units of meaning. In the analyses, we used Systematic Text Condensation and an editing analysis style [29]. Two researchers (GR and KH) independently read all the material searching for an overall impression and established preliminary subthemes. The rest of the group read one interview each, gave input, and wrote a summary of the overall impression of the interview. We then examined the text for units of meaning representing information about students’ experiences. In an iterative process, we coded and grouped these units, contrasted and abstracted the content in each group, and finally discussed and summarized the content of each group into generalized descriptions. To support the analysis, we used field notes and created mind maps, and discussed the analysis at each step to reach an agreement. Quotations were used to illustrate and support findings.

### User involvement

A second-year student (MM) took part in the design and guided questions for the study. This student was also a part of the research group preparing the manuscript.

### Ethics

The study was conducted according to research ethics guidelines in accordance with the Helsinki Declaration. Application was approved by the Head of Department at the University, the Norwegian Center for Research Data (Project No 973745), and the research ethics committee at the Faculty (FEK). The students received oral and written information about the study and gave their written consent to participation.

## Results

### Quantitative data

Three hundred and ninety-six baccalaureate nursing students (response rate, 46%) at University of Agder participated in the survey. The characteristics of the students are presented in Table 1. The majority (70%) of students were below 25 years of age, were living with someone (84%), had felt lonely due to the pandemic (56%), and had been engaged in clinical placement during the pandemic (62%) (Table 1). Most students (72%) had trust in the government’s handling of the COVID-19 situation; the same was the case for the universities’ handling of the situation (57%). Moreover, 70% of the students were concerned about the quality of education they were receiving (Table 1).

**Table 1:** Characteristics of the baccalaureate nursing students at University of Agder (*N* = 396) organized by school year

	Total N = 396	Year 1 *N* = 142	Year 2 *N* = 127	Year 3 N = 127	*P*-values*
Years in nursing school					
1 2 3	142 (36%) 127 (32%) 127 (32%)				
Age, years					
< 25 25–29 ≥ 30	278 (70%) 46 (12%) 72 (18%)	112 (79%) 11 (8%) 19 (13%)	86 (65) 26 (20%) 15 (12%)	80 (63%) 20 (16%) 27 (21%)	0.056
Living alone					
No Yes	332 (84%) 64 (16%)	120 (85%) 22 (15%)	108 (85%) 19 (15%)	104 (82%) 23 (18%)	0.764
Number of times tested for COVID-19					
Never 1 2 3 ≥ 4	154 (39%) 124 (31%) 60 (15%) 40 (10%) 18 (5%)	58 (41%) 44 (31%) 15 (11%) 16 (11%) 9 (6%)	46 (36%) 42 (33%) 27 (21%) 10 (8%) 2 (2%)	50 (39%) 38 (30%) 18 (14%) 14 (11%) 7 (6%)	0.237
Quarantine status related to COVID-19					
Never Previous Now	236 (60%) 157 (39%) 3 (1%)	77(54%) 63 (44%) 2 (2%)	78 (61%) 48 (38%) 1 (1%)	81 (64%) 46 (36%) 0	0.383
At risk for COVID-19 complications					
No Uncertain Yes	49 (10%) 323 (82%) 33 (8%)	16 (11%) 114 (80%) 12 (9%)	16 (13%) 103 (81%) 8 (6%)	8 (6%) 106 (84%) 13 (10%)	0.397
Trust in governmental handling of the COVID-19 situation					
Strongly disagree/disagree Neither disagree nor agree Agree Strongly agree	36 (9%) 44 (18%) 200 (50%) 89 (22%)	14 (6%) 30 (21%) 67 (47%) 31 (22%)	4 (3%) 29 (23%) 68 (53%) 26 (20%)	18 (14%) 12 (9%) 65 (51%) 32 (25%)	0.016
Trust in universities’ handling of the COVID-19 situation					
Strongly disagree Disagree Neither disagree nor agree Agree Strongly agree	18 (4%) 44 (11%) 112 (28%) 180 (46%) 42 (11%)	5 (4%) 17 (12%) 37 (26%) 67 (47%) 16 (11%)	4 (3%) 17 (13%) 47 (37%) 50 (39%) 28 (22%)	9 (7%) 10 (8%) 28 (22%) 63 (50%) 17 (13%)	0.080
Concern about the quality of education					
Strongly disagree Disagree Neither disagree nor agree Agree Strongly agree	22 (5%) 34 (9%) 64 (16%) 142 (36%) 134 (34%)	3 (2%) 6 (4%) 17 (12%) 50 (35%) 66 (47%)	6 (5%) 13 (10%) 21 (17%) 40 (31%) 47 (37%)	13 (10%) 15 (12%) 26 (21%) 52 (41%) 21 (16%)	< 0.001
Fear of COVID-19 (Mean (SD))	2.3 (0.7)	2.5 (0.8)	2.3 (0.7)	2.2 (0.7)	0.026
Feeling lonely due to COVID-19					
Strongly disagree Disagree Neither disagree nor agree Agree Strongly agree	33 (8%) 61 (15%) 80 (20%) 130 (32%) 92 (23%)	8 (6%) 16 (11%) 23 (16%) 53 (37%) 42 (30%)	10 (8%) 21 (16%) 28 (22%) 40 (32%) 28 (22%)	15 (12%) 24 (19%) 29 (23%) 37 (29%) 22 (17%)	0.096
Engagement in clinical practice during the pandemic					
Yes No	246 (62%) 150 (38%)	12 (9%) 130 (91%)	117 (92%) 10 (8%)	127 (92%) 10 (8%)	< 0.001
Have you during the pandemic been in contact with patients with the following situation?					
Patients with unclear COVID-19 status Patients with confirmed COVID-19 infection Both (unclear and/or confirmed) None of them	148 (61%) 7 (3%) 35 (15%) 52 (21%)	3 1 0 6	64 (55%) 2 (2%) 11 (9%) 39 (34%)	81 (70%) 4 (3%) 24 (21%) 7 (6%)	< 0.001
Self-imposed quarantine during clinical practice					
Yes No	105 (43%) 137 (57%)	2 8	43 (37%) 73 (63%)	60 (52%) 56 (48%)	0.025

*Categorical data are presented as number (%) and continuous variables as mean (SD). Chi-square tests were used to compare differences in categorical variables and ANOVA tests for continuous data

When comparing students in the three different years, the most striking difference was that most first-year students had not been engaged in clinical placement during the pandemic by February 2021. This difference influenced on more second-year (66%) and third-year students (94%) who had been in contact with patients with confirmed or unclear COVID-19 status compared to first-year students. (Table 1).

For students engaged in clinical practice (second- and third-year students), 74% reported having sufficient knowledge and skills to handle infection control due to COVID-19, 69% were worried about contracting COVID-19, and 85% were worried about spreading the virus to patients (Table 2). Furthermore, 81% of the students were worried about too much sick (or quarantine) leave due to the required number of days to pass clinical studies, 88% were worried about not being able to finish clinical studies, 50% experienced reduced learning situations, and 25% reported insufficient guidance (see details in Table 2). There were no significant differences between second- and third-year students.

**Table 2:** Learnings outcomes in clinical practice of second- and third-year (*N* = 254) baccalaureate nursing students at University of Agder

	Necessary knowledge of infection control	Concerns about getting infected during clinical practice	Concerns about infecting patients during clinical practice	Concerns about high absenteeism during clinical practice	Concerns about completion of clinical practice	Fewer learning situations during clinical practice	Insufficient guidance during clinical practice
Strongly disagree	4 (2%)	14 (6%)	6 (2%)	14 (6%)	8 (3%)	34 (14%)	54 (22%)
Disagree	20 (8%)	30 (12%)	23 (10%)	12 (5%)	8 (3%)	46 (19%)	84 (35%)
Neither disagree nor agree	39 (16%)	31 (13%)	7 (3%)	19 (8%)	14 (6%)	39 (16%)	45 (18%)
Agree	127 (53%)	98 (40%)	87 (36%)	61 (25%)	67 (28%)	76 (31%)	38 (16%)
Strongly agree	52 (21%)	69 (29%)	119 (49%)	136 (56%)	145 (60%)	47 (19%)	21 (9%)

No significant differences between year 2 and year 3 students were identified

### Qualitative data

**Fig. 1:**
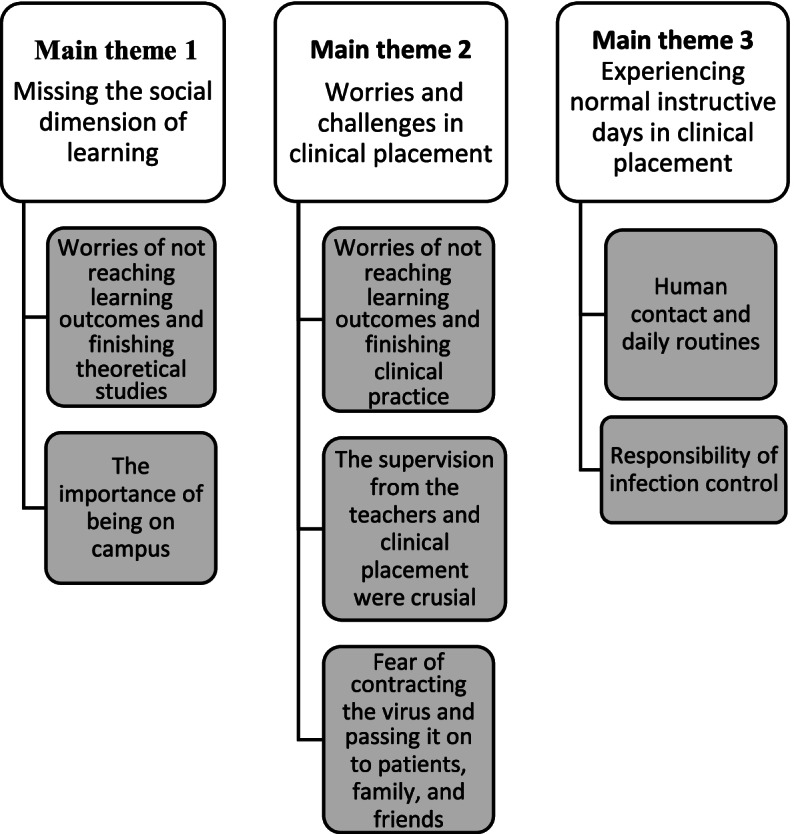
Main themes and subthemes of how the covid 19 pandemic influenced baccalaureate nursing students’ experiences of learning

Among the 23 students who took part in one of the five focus groups, the age range was 19–32 years, with a mean age of 23 years. The groups comprised 10 first-year students, seven second-year students, and six third-year students. All students (except 1) were living in shared accommodation or with a partner. During the analyses of the qualitative data, we identified three main themes: 1. Missing the social dimension of learning; 2. Worries and challenges in clinical placement; and 3. Experiencing normal instructive days in clinical placement*.* Each theme comprised two or three subthemes, illustrated in Fig. 1.

### Missing the social dimension of learning

Due to COVID restrictions, most tutorials and classes on campus were digital, using different platforms such as streaming, Teams, and Zoom. Most students missed the on-campus tutorials. The digital tutorials required more discipline from students, which some of them admitted could have been better maintained. The continuity towards learning outcomes, goals, and the flow in the classes could be hard to see or follow due to digital lessons and limited dialogue. The students were worried about not reaching their learning outcomes and not being able to finish their studies. This was especially prominent among first-year students. The quality of the digital tutorials varied, boring videos implied decreased motivation, and the students preferred digital platforms like Zoom, with the possibility for discussions. The students also noticed that most professors became more familiar with the digital teaching as time went by.

The students missed face-to-face discussions and dialogue with fellow students and the professors. They missed the opportunity to ask questions and to get to know other students and the professors. This was especially true among first-year students. Some first-year students felt alone and knew only a few fellow students, admitting it was hard to take the initiative to get to know fellow students better. Two students considered leaving the university or starting again after the pandemic. Furthermore, students with learning disability found it hard not to be able to see the professors in person and ask questions when needed.

“You get a lot of free learning from going to campus. You may ask your fellow students or your professor if you did not understand something, and then maybe you start a discussion in a group. This is something you don’t get the opportunity to do now.” (Focus group nr 3)

To get to know fellow students and the professors was considered important for optimal learning. The digital solutions made it hard to meet fellow students, and it was difficult to establish new friendships. The social part of being a student and the possibility to see other students was emphasized as important for student learning and well-being. The students’ motivation for learning decreased, so did their feeling of university belonging.

“You don’t belong. You lose your sense of purpose.” (Focus group nr 5*)*

Second- and third-year students underlined the importance of the “free learning” they received when they had the opportunity to attend campus, ask questions, discuss, and revise a lesson. Students with an established network felt more privileged than first-year students. Some students went to the university library just to watch digital tutorials and meet a few fellow students during breaks. One of the year 2 students said,

“I am very happy that I was in year 2 and not in year 1 when the society went into lockdown.” (Focus group nr 2)

### Worries and challenges in clinical placement

In contrast to most other universities, the students at the present university were able to attend clinical placements nearly as much as before the pandemic, and they really appreciated it. Although the students were able to attend clinical practice, they were worried about not reaching learning outcomes. The students were worried about not being able to fulfill the number of hours and days required in clinical studies and afraid of not qualifying for nursing registration. A COVID-19 infection or period of quarantine could easily cause such a situation.

“It is not just the fear of catching the virus or spreading the virus but also the fear of the consequences of a positive test and the quarantine. Do I have to postpone my authorization as a nurse due to lack of clinical practice?” (Focus group nr 5)

Some students did not meet some of the required learning outcomes, while others’ experience was to reach them satisfactorily. It had recently been a discussion among the students about a generation of nurses receiving a second-best education due to lack of experience and learning; however, most of the students in the present study did not feel so.

“There are several nursing students at other universities in the country who are going to finish their studies this spring who feel they will end as a second-best nurse. This is not the case for us. I feel like a trained and proper nurse.” (Focus group nr 2)

The students had to adapt to the national restrictions and rules, which constantly varied during the first year of the pandemic due to transmission rate and number of vaccinated patients and staff. The guidelines from the university were unclear, especially in the beginning of the pandemic. A few students experienced a placement that did not want students due to work overload and unclear situations, although most students were satisfied with their supervisors who had walked the extra mile to assist them.

Fear of catching the virus themselves or spreading the virus to patients, family, and fellow students when in clinical studies was emphasized by all students. Several nursing homes in the region had experienced COVID-19 infections among patients and staff, and recently a nursing student had been responsible for bringing the infection into the ward. They were afraid of being the student who brings the infection and causes death.

“I can imagine the newspaper; nursing student brought the virus into the nursing home and 5 patients died.” (Focus group nr 4)

As a result of contact with a high number of persons in clinical practice, the students reduced the number of persons they met in their spare time.

### Experiencing normal instructive days in clinical placement

The students found that clinical practice brought routine into their lives. They were practicing meaningful newly-learned skills. Especially, the first-year students felt that they finally were able to have a more normal life. They experienced having a routine, a social life of sorts, and colleagues. In clinical practice, several of the students had met COVID-19-infected patients and patients with unclear COVID-19 status. In the emergency ward, some had been in the frontline and seen how severely ill the patients could be. Furthermore, they experienced all the extra precautions and the extra work this entailed. They witnessed the nurses’ extra workload, working double shifts to cover up and taking care of lonely patients. As a result of these experiences, they felt an extra responsibility to take preventive action to not spread the virus.

“I was in the frontline at the emergency ward and saw how severely ill the patients could be.” (Focus group nr 4)

Furthermore, as nursing students they were expected to have more than average knowledge about hygiene and infection control. They felt responsibilities to act as role models. One student highlighted this and said,

“I remember my own attitude to hygiene before the corona. I could be sloppy sometimes. But now I wash my hands all the time. So, I have developed increased awareness in hygiene.” (Focus group nr 4)

Being “inside” the pandemic brought new perspectives. The students had experienced the overwhelming workload within the health care system through the pandemic, gaining a perspective that other students may miss.

## Discussion

The aim of this study was to explore how the COVID-19 pandemic influenced baccalaureate nursing students’ experiences of learning. The general picture that emerges from our analysis is the importance of social interactions for learning [20]. The students were worried about not reaching learning outcomes and had worries and challenges in clinical placement. However, they experienced more normal days in clinical placement and a feeling of being a part of the history.

The nursing students experienced a fear of not reaching learning outcomes both in theoretical classes and clinical practice because of all the adaptations and restrictions imposed due to the COVID-19 pandemic. These findings are in line with previous studies where nursing students had problems with concentration and learning efficiency, fear of COVID-19, their grades, final graduation, and future career [11, 14, 18]. Looking closer into the numbers in the national survey [23], more students in the present university reported satisfaction with the university’s handling of the situation and were less concerned about the quality of the education compared with other universities. Such findings were most likely associated with the relatively low transmission rate during the pandemic and local restrictions in the university region [21], along with nearly normal maintenance of clinical practice, which was not seen in the other universities [5]. Despite this, our students experienced fear of not reaching their learning outcomes, experienced stress, missed their peers and friends, and experienced fear of COVID.

The fear of COVID and its concomitant restrictions hampered the possibilities and advantages that social interactions bring to learning [20]. A recent study from Sweden [30] showed that while some of the nursing students preferred digital learning and use of digital tools, the majority said that digital learning reduced their opportunities for social interaction and felt that this reflected negatively on their learning process. Moreover, students reported that the reduced social interaction had a negative effect on their mental health [30]. In most nursing education, learning is regarded as a social experience rather than an isolated one, and social interaction is a significant element in the learning process [20]. Reduced social interaction affects both the learning process and the students’ mental health. The results from our study showed that first-year students were more vulnerable than second- and third-year students. They felt lonely and some of them had few fellow students. The pandemic forced the professors to start with digital teaching instead of traditional campus-based teaching, and most of the professors were not prepared for this. A longer preparation time might have resulted in the development of learning activities that better supported social interaction, such as digital groups and discussion [8]. Although the students in our study were able to attend clinical practice, they were worried about not reaching expected learning outcomes and not being able to fulfill the number of days required in clinical studies.

Both the quantitative and qualitative data show that first-year students are more vulnerable than second- and third-year students. This might be because first-year students have not yet been able to meet each other face-to-face, are not used to being tertiary students, and are strongly affected by the uncertainty of the future. Already at the beginning of the nursing program, they were worried about not being able to complete their education and become a nurse. Living with such uncertainty and stress may have a negative impact on the learning process [14, 15, 18].

Despite the nursing students’ fear and struggles, they were also grateful for the experience of being a part of a historical tradition**.** Some studies show that the status of nursing has risen during the pandemic [31]. Our findings are in line with those of Swift et al. [17] who showed that where students successfully stepped up to a learning challenge, they found it both personally fulfilling and professionally worthwhile. Such experiences were mostly related to the students’ clinical placement, where several students in our study told stories about how they had become “a positive difference” for the patients and in the ward.

### Strengths and limitations

Strengths of the study are that nearly half of the students at the present university participated in the cross-sectional survey, a response rate considered to be acceptable in surveys like this. Furthermore, the proportion of students in the three different years was close to the same. Quantitative data were collected using well-validated questions and questionnaires, most of them used in previous student surveys in Norway [5].

Limitations with the present study are the cross-sectional nature of the survey, which reveals only statistically significant associations between the variables and does not allow one to draw conclusions about causality. We have no information about the students not taking part in the survey. However, quantitative results were combined with qualitative findings to give a more comprehensive picture of the students’ experiences, and adds valuable informations.

### Implications

It is of great importance for most of the students that their life is predictable, and they have a sense of purpose through their years as a student, despite a pandemic. This becomes extremely important for first-year students. The results show that the social learning environment in the future should be safeguarded by offering both digital and physical teaching (lectures and simulation) on campus. Moreover, the results emphasized the importance of social interactions for learning, and that the universities need to develop strategies that compensate for the lack of social interaction to improve the students’ well-being and psychosocial health.

Clinical placement met students’ needs for social and academic meetings, as well as individual meetings with professors, and must be preserved and strengthened as a learning forum in future pandemics and other demanding situations. Furthermore, professors must maintain the ability to think of alternative learning strategies that can be implemented quickly in the event of future pandemics (or other crises).

## Conclusion

The COVID-19 pandemic influenced nursing students’ learning significantly. Most of the students were concerned about the quality of their education and were worried about excessive absence from practical placements due to COVID infection or quarantine. The students missed their face-to-face contact with their peers and professors, which underlined the importance of social interaction for learning. Despite challenges in clinical practice, the students appreciated the experience of being a part of history. The challenges of the students’ experiences must be incorporated into the university’s curriculum and teaching.

## Supplementary Information

**Additional file 1.**

## Data Availability

The datasets used and/or analyzed during the current study are not publicly available due to General Data Protection Regulation laws but are available from the corresponding author on reasonable request and with permission from the Norwegian Centre for Research Data.
