# Supplementary material for: Baccalaureate nursing students’ experiences of how the COVID-19 pandemic influenced learning – a mixed method study
Source: BMC Nurs. 2022 Jul 1;21:172. doi: 10.1186/s12912-022-00955-6 (PMC9247899; doi:10.1186/s12912-022-00955-6)
Supplement: Supplementary file 1 — Additional file 1. [file 12912_2022_955_MOESM1_ESM.docx]

Supplementary Table 1

Characteristics of the baccalaureate nursing students at University of Agder (N = 396) and four other universities

|  | University of Agder  N = 396 | Other universities  N = 2310 | *P*-value |
| --- | --- | --- | --- |
| Years in nursing school    1    2    3 | 142 (36%)  127 (32%)  127 (32%) | 933 (42%)  674 (31%)  603 (27%) | 0.043 |
| Age, years    < 25    25–29    ≥ 30 | 278 (70%)  46 (12%)  72 (18%) | 1567 (71%)  551 (15%)  310 (14%) | 0.037 |
| Living alone    No    Yes | 332 (84%)  64 (16%) | 1797 (82%)  403 (18%) | 0.179 |
| Number of times tested for COVID-19    Never    1    2    3    ≥ 4 | 154 (39%)  124 (31%)  60 (15%)  40 (10%)  18 (5%) | 661 (27%)  600 (21%)  385 (17%)  286 (13%)  328 (15%) | < 0.001 |
| History of positive COVID-19 test    No    Yes | 381 (96%)  15 (4%) | 2100 (95%)  110 (5%) | 0.422 |
| Quarantine status related to COVID-19    Never    Previous    Now | 236 (60%)  157 (39%)  3 (1%) | 1066 (48%)  1098 (50%)  46 (2%) | < 0.001 |
| At risk for COVID-19 complications    No    Uncertain    Yes | 40 (10%)  323 (82%)  33 (8%) | 287 (13%)  1766 (80%)  156 (8%) | 0.213 |
| Trust in governmental handling of the COVID-19 situation    Strongly disagree/disagree    Neither disagree nor agree    Agree    Strongly agree | 36 (9%)  71 (18%)  200 (51%)  89 (22%) | 195 (9%)  491 (22%)  1144 (52%)  380 (17%) | 0.080 |
| Trust in universities’ handling of the COVID-19 situation    Strongly disagree    Disagree    Neither disagree nor agree    Agree    Strongly agree | 18 (4%)  44 (11%)  112 (28%)  180 (56%)  42 (11%) | 163 (7%)  403 (18%)  671 (30%)  802 (36%)  171 (8%) | < 0.001 |
| Concern about the quality of education    Strongly disagree    Disagree    Neither disagree nor agree    Agree    Strongly agree | 22 (5%)  34 (9%)  64 (16%)  142 (36%)  134 (34%) | 72 (3%)  (126 (6%)  223 (10%)  727 (33%)  1062 (48%) | < 0.001 |
| Feeling lonely due to COVID-19    Strongly disagree    Disagree    Neither disagree nor agree    Agree    Strongly agree | 33 (8%)  61 (16%)  80 (20%)  130 (33%)  92 (23%) | 131 (6%)  319 (14%)  364 (16%)  769 (35%)  626 (28%) | 0.048 |
| Engagement in clinical practice during the pandemic  Yes    No | 246 (62%)  150 (38%) | 1345 (61%)  864 (39%) | 0.343 |
| Have you during the pandemic been in contact with patients with the following situation?    Patients with unclear COVID-19 status    Patients with confirmed COVID-19 infection    Both (unclear and/or confirmed)    None of them | 146 (61%)  7 (3%)  35 (15%)  52 (21%) | 664 (51%)  44 (4%)  203 (15%)  389 (30%) | 0.017 |
| Fear of Covid-19 | 2.3 (0.7) | 2.5 (0.8) | < 0.001 |

The number of students will vary because some students lack clinical practice during the pandemic period.

Supplementary Table 2

Learning outcomes in clinical practice of second- and third-year students (N = 1553) at University of Agder (N = 242) and four other universities (N = 1311)

|  | University of Agder N = 242 | | | | | Other universities N = 1311 | | | | | *P*-value* |
| --- | --- | --- | --- | --- | --- | --- | --- | --- | --- | --- | --- |
|  | Strongly  disagree | Disagree | Neither disagree nor  agree | Agree | Strongly  agree | Strongly  disagree | Disagree | Neither disagree nor  agree | Agree | Strongly  agree |  |
| Necessary knowledge of infection control | 4 (2%) | 20 (8%) | 39 (16%) | 127 (52%) | 52 (22%) | 15 (1%) | 66 (5%) | 183 (14%) | 753 (56%) | 315 (24%) | 0.205 |
| Concerns about getting infected during clinical practice | 14 (6%) | 30 (12%) | 31 (13%) | 98 (49%) | 69 (29%) | 100 (7%) | 219 (17%) | 206 (16%) | 457 (35%) | 329 (25%) | 0.126 |
| Concerns about infecting patients during clinical practice | 6 (3%) | 23 (9%) | 7 (3%) | 87 (36%) | 119 (49%) | 39 (3%) | 55 (4%) | 87 (7%) | 461 (35%) | 669 (51%) | 0.002 |
| Concerns about high absenteeism during clinical practice | 14 (6%) | 12 (5%) | 19 (8%) | 61 (25%) | 136 (56%) | 54 /4%) | 107 (8%) | 119 (9%) | 357 (27%) | 674 52%) | 0.232 |
| Concerns about completion of clinical practice | 8 (3%) | 8 (3%) | 14 (6%) | 67 (28%) | 145 (60%) | 22 (2%) | 42 (3%) | 79 (6%) | 378 (29%) | 790 (60%) | 0.570 |
| Fewer learning situations during clinical practice | 35 (14%) | 46 (19%) | 39 (16%) | 76 (31%) | 47 (20%) | 107 (8%) | 169 (13%) | 229 (15%) | 376 (29%) | 429 (33%) | < 0.001 |
| Insufficient guidance during clinical practice | 54 (22%) | 85 (35%) | 45 (18%) | 38 (16%) | 21 (9%) | 219 (17%) | 350 (27%) | 351 (27%) | 364 (17%) | 185 (12%) | 0.002 |

*Chi-square test was used to compare students at University of Agder with the four other universities.
